# Influence of weather and atmospheric pollution on physical activity in patients with COPD

**DOI:** 10.1186/s12931-015-0229-z

**Published:** 2015-06-13

**Authors:** Ayedh D. Alahmari, Alex J. Mackay, Anant R.C. Patel, Beverly S. Kowlessar, Richa Singh, Simon E. Brill, James P. Allinson, Jadwiga A. Wedzicha, Gavin C. Donaldson

**Affiliations:** Centre for Respiratory Medicine, University College London, Royal Free Campus, Rowland Hill Street, Hampstead, NW3 2PF London, UK; UK Airways Disease Section, National Heart and Lung Institute, Imperial College London, Emmanuel Kaye Building, London, UK

**Keywords:** COPD, Atmospheric pollution, Weather, Daily step-count, Physical activity, Daily monitoring

## Abstract

**Rationale:**

Information concerning how climate and atmospheric pollutants affects physical activity in COPD patients is lacking and might be valuable in determining when physical activity should be encouraged.

**Methods:**

Seventy-three stable COPD patients recorded on daily diary cards worsening of respiratory symptoms, peak expiratory flow rate, hours spent outside the home and the number of steps taken per day. Pedometry data was recorded on 16,478 days, an average of 267 days per patient (range 29-658). Daily data for atmospheric PM_10_ and ozone (O_3_) were obtained for Bloomsbury Square, Central London from the Air Quality Information Archive databases. Daily weather data were obtained for London Heathrow from the British Atmospheric Data Archive.

**Results:**

Colder weather below 22.5 °C, reduced daily step count by 43.3 steps day per°C (95 % CI 2.14 to 84.4; *p* = 0.039) and activity was lower on rainy than dry days (*p* = 0.002) and on overcast compared to sunny days (*p* < 0.001). Daily step count was 434 steps per day lower on Sunday than Saturday (*p* < 0.001) and 353 steps per day lower on Saturday than Friday (*p* < 0.001). After allowance for these effects, higher O_3_ levels decreased activity during the whole week (-8 steps/ug/m3; *p* = 0.005) and at weekends (-7.8 steps/ug/m3; *p* = 0.032). Whilst, during the week PM_10_ reduced activity (*p* = 0.018) but not during the weekend.

**Conclusions:**

Inactivity of COPD patients is greatest on cold, wet and overcast days and at the weekends. This study also provides evidence of an independent effect of atmospheric pollution at high levels.

**Electronic supplementary material:**

The online version of this article (doi:10.1186/s12931-015-0229-z) contains supplementary material, which is available to authorized users.

## Introduction

Chronic obstructive pulmonary disease (COPD) causes much morbidity and reduces quality of life [1]. The disease is projected to become the fourth leading cause of death worldwide by 2030 [2]. COPD involves airflow obstruction that results in dyspnoea which is associated with reduced daily activity and increased muscle weakness [3].

Patients with COPD experience episodes of acute worsening of in their respiratory symptoms termed exacerbations that are often triggered by respiratory infection [1, 4]. Frequent exacerbations have an accelerated decline in lung function [5] and an increase rise in airway and systemic inflammation [6, 7]. Patients with frequent exacerbations patients also becoming housebound faster [8] and have greater perception of fatigue [9] which might explain why this group suffers more from depression [10]. We have previously reported that physical activity is reduced during a COPD exacerbation [11]. In this study, we report only on data collected when the patients were clinically stable.

A few studies have examined activity in older people [12, 13] but we are unaware of any studies that have specifically examined the impact of daily weather on physical activity in patients with COPD.

Pollution may also reduce activity and we have previously reported that particulate matter <10 μm in diameter (PM_10_) in London increases symptoms of dyspnoea in COPD patients [14] and reduce pulmonary function [15]. Traffic-related air pollution exposure has also been shown to be positively associated with first hospital admission for COPD [16].

Maintenance of physical activity can substantially reduce age-related mortality [12] but it is particularly important for patients with COPD since those who continued to exercise have less dyspnoea, fewer hospital admissions for COPD and reduced mortality. Indeed, COPD patients can be referred by physicians to specialized pulmonary rehabilitation clinics to undergo a few weeks of physical training and education but poor participation and a failure to continue exercising limits the effectiveness of this intervention. There is a need therefore to understand the barriers to participation and sustained behaviour change [17]. In this study, we examine for the first time in patients with COPD how the weather and atmospheric pollution levels affect physical activity.

## Methods

### Patient recruitment

The London COPD cohort is a group of approximately 200 COPD patients under longitudinal observation at the Centre for Respiratory Medicine, University College London. This cohort was started in 1995 for the prospective investigation of COPD exacerbations. Patients who withdraw or die are replaced on a rolling basis. COPD is defined as a Forced Expiratory Volume in 1 s (FEV_1_) ≤ 80 % of a normal value predicted from age, height, and sex and a FEV_1_/Forced Vital Capacity (FVC) ratio < 0.7. Patients enrolled in the cohort complete daily diary cards and are seen in clinic every 3 months if stable and annually undergo a comprehensive medical review. Patients were also seen at exacerbation and most were prescribed oral corticosteroids and/or antibiotics. Patients with any other primary respiratory diseases or who are unable or unwilling to complete daily diary cards were excluded.

In April 2011, there were 199 patients enrolled in the London COPD cohort. 24 patients were ineligible as they used a walking support (cane or frame) or were confined to a wheel chair or used ambulatory oxygen cylinders and 30 refused. We eventually provided pedometers to 145 patients. Data was successfully acquired from only 73 patients for the following reasons a) 21 patients once issued refused to use the pedometer, b) 19 patients lost their pedometers, c) 23 patients recorded less than 35 days of data whilst stable due to repeated exacerbation and, d) 9 pedometers malfunctioned. The study ended in March 2013.

A full medical and smoking history was taken and measurements of FEV_1_ and FVC made with a Vitalograph Gold Standard spirometer (Vitalograph Ltd, Maids Moreton, UK). Body mass index (BMI) was calculated from height and weight.

#### Monitoring

Patients were educated to use diary cards at the recruitment visit and re-educated as needed when visiting the clinic. The diary cards also have instructions (how to fill the card and how use the pedometer) and contact numbers on the back of every card. All patients kept a daily diary card on which they recorded any worsening in their respiratory symptoms, the number of hours spent outside their home and their daily peak expiratory flow (PEF) measured with a mini-Wright meter (Clement-Clark International, Harlow, UK) once a day at morning. Patients were instructed to wear a pedometer (Yamax Digi-Walker SW-200) on left side of body all the time, except when sleeping or showering. Patients recorded daily step counts on written daily diary cards. This pedometer has been shown to accurately measure steps in free-living individuals [18, 19] and in normal and moderately obese patients and [20] detected differences in physical activity of COPD patients [21]. Patient also completed a daily COPD Assessment Test (CAT) questionnaire after first being trained in clinic. Pedometry data collected over the initial 7 days were discarded to avoid any learning effects and only patients who had recorded more than 35 days of data were included in this analysis.

#### Exacerbations

Exacerbations were identified according to our usual criteria of increases in any two major symptoms (dyspnoea, sputum volume or sputum purulence) or one major and one minor symptom (nasal congestion, wheeze, cough, sore throat) over two consecutive days [22]. Data recorded two weeks either side of the onset of an exacerbation were excluded from the analysis.

#### Ethics

The study was approved by the London-Hampstead research ethics committee and all patients gave written informed consent (REC 09/H0720/8).

#### Temperature and pollution data

Daily data for atmospheric PM_10_ and ozone (O_3_) were obtained for Bloomsbury Square, Central London from the Air Quality Information Archive databases (http://www.airquality.co.uk). Data from the archive is reported as μg/m^3^. The conversion factor of ozone is 1 ppb = 1.9957 μg/m^3^ at 20 °C and 1013 millibar atmospheric pressure. We did not use data from the monitoring site in Hackney that would be closer to our patients because it did not record data on PM_10_ which we have previously shown to increase dyspnoea [14].

Weather data was the average of hourly readings over 24 h at Heathrow Airport and obtained from the British Atmospheric Data Centre (www.badc.nerc.ac.uk). A dry day was defined as zero precipitation [23] and a sunny day arbitrarily defined as a day when the sun shone for a minimum 0.1 h or more.

#### Statistical methods

Patient characteristics are summarised as appropriate by a mean and standard deviations or standard errors, or a median and inter-quartile ranges, or as a percentage.

#### Unadjusted analysis

Generalised estimating equations (GEE) were used to model the effects of weather and pollution on daily step count, PEF, CAT scores (assuming their Gaussian distribution), time (hours) outdoors (as Poisson distributed) or worsened dyspnoea (with a Bernoulli distribution) on days with temperatures ≤22.5 °C. It was an a priori decision that a cut-off would be necessary as relatively hot weather can reduce time spent outdoors [24]. To identify the inflexion in the relationship between activity and temperature we plotted mean daily step count against temperature in 0.25 °C intervals. After inspection, a cut-off of 22.5 °C was chosen as daily step count was highest at this temperature and decreased with temperatures below or above 22.5 °C. GEE models were used to examine our panel data as they correct the standard errors and p-values for the various regression coefficients for the correlation structure between the repeated measurements on the same patient. We used the xtgee command in Stata with the robust option as this would produce valid standard errors even if our assumption of an independent correlation structure was incorrect.

Comparisons between daily step count of a sunny compared to a dull day, or a dry versus rainy day were made by paired *t*-test, after first obtaining the average for each patient under the various conditions.

Analysis of variance (ANOVA) was used to determine the effect of day of the week on daily step count, hours outdoors, O_3_ and PM_10_. Post-hoc comparisons were made between Sunday and Saturday, and between Saturday and Friday.

#### Adjusted analysis

GEE regression models were used to assess the independent effects of climate and pollution on daily step count and the other outcome measures. These models included a linear term to adjust for age related decline, sine and cosine terms with periods of 12, 6 and 4 months to allow for seasonal changes, and a variable for day of week with Monday as the first day of the week. The covariates also included daily temperature, wind speed, rainfall, hours of sunshine and day-length, PM_10_ and O_3_ as independent variables. We did not examine any lagged effects of climate or pollution. The analysis was repeated using data collected during week-days only (Monday-Friday) and during week-ends (Saturday and Sunday) since activity was markedly dissimilar in these periods. The analysis was also repeated with an auto-regressive term (the previous day value of the dependent variable) in the model to adjust for autocorrelation in the dependent variable. We also repeated the analysis of daily step count and the pollutants with time outdoors included as an independent variable.

#### Distance to pollution monitoring site

The coordinates of Bloomsbury Squares and the patient’s home (defined as the centre of their post-code) were obtained from the National Statistics postcode directory database. The straight-line distance between the two sets of coordinates was calculated by Pythogras’ theorem.

## Results

### Patient characteristics

The 73 COPD patients (51 Male, 22 Female) studied had moderate to very severe COPD (Table 1). There were no significant differences in the patient characteristics between the 73 patients involved in this study and 126 patients excluded for reasons described in the methods. The patients recorded daily step count on 16,478 days with an average per patient of 267 days (range 29-658). Of these, 3020 days were excluded, as exacerbations commenced either two weeks before or after.

**Table 1:** Characteristics of the 73 COPD patients in the study and 126 COPD patients in the London COPD Cohort not recruited to the study

	Recruited COPD patients (n = 73)	Not recruited COPD patients (n = 126)	*P-value*
	*Mean (±SD)*	*Mean (±SD)*	
Age (years)	71.1 (±8.7)	70.2 (±8.8)	0.51
FEV_1_ (l)	1.31 (±0.5)	1.40 (±0.5)	0.25
FEV_1_ (% predicted)	52.9 (±16.5)	56.2 (±16.1)	0.22
FVC (l)	2.79 (±0.9)	2.76 (±0.9)	0.83
FEV_1_/FVC (%)	47.8 (±12.6)	50.7 (±12.3)	0.11
BMI (kg/m^2^)	26.8 (±5.6)	27.0 (±5.1)	
	*Median (IQR)*	*Median (IQR)*	
Exacerbations/year	2% (1.0-3.0)	1.4 (0.7-3.0)	0.40
Sex (Males)	69.9	62.6	0.36
Chronic bronchitis	54.3	54.5	0.98
Smoking at recruitment	35.6	32.0	0.77

The patients lived on average 7.39 km (SD 4.70) from the Bloomsbury Square site. Of the 73 patients, 59 lived north-east, 7 north-west, 2 south-east and 5 south-west of Bloomsbury.

**Fig. 1:**
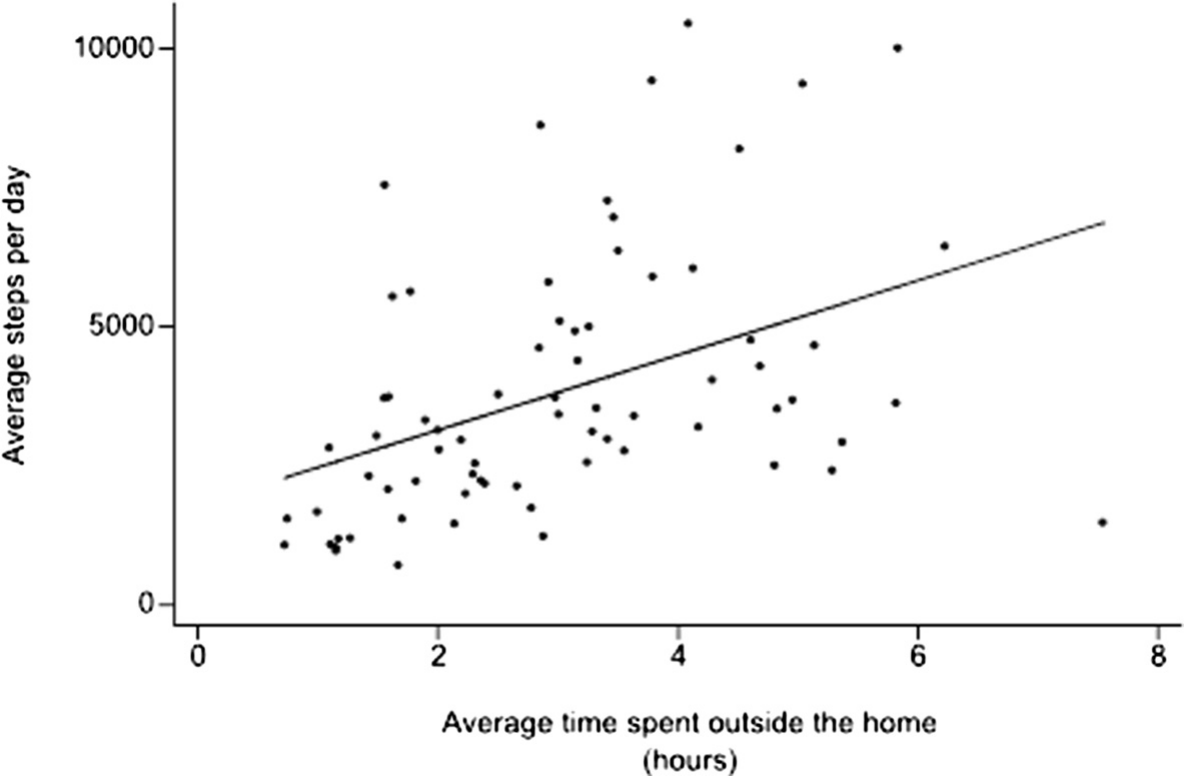
Relationship between the average steps per day for each patient and the average hours spent outside the home during the whole week

The average of 225 days of pedometry readings per patient (SD 139; range 29-578); 459 days of PEFR readings per patient (SD 139; range 124-768); 463 days per patient of whether or not dyspnoea was worse than usual (SD 138; range 124-680) and 70 days with a CAT score per patient (SD 87; range 0-356). During the week, when patients might be at work, the mean of each patient’s average time outside the home per day was 3.05 h (SD 1.51; range 0.52 to 7.3 h). Over the whole week, there was a strong relationship between the average number of steps per day and the average time spent outdoors (regression coefficient =671 steps per day per hour outdoors; intercept = 1804 steps per day; *p* = 0.001; see Fig. 1).

### Unadjusted analysis

**Fig. 2:**
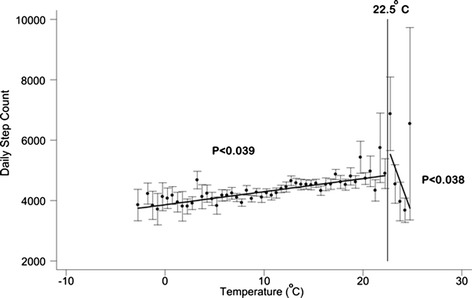
Relationship between daily step count and daily temperature; data is averaged in 1 °C intervals. Bars are standard errors

Warmer weather was associated with increased daily step count (Fig. 2). A 1 °C rise in temperature increased the count by 43 steps per day per °C (95 % CI 2.14 to 84.4; *p* = 0.039). However, when the temperatures exceeded 22.5 °C, patient activity appeared to decrease and steps per day fell by -891 per 1 °C rise (95 % CI -1735 to -47; *p* = 0.038).

**Fig. 3:**
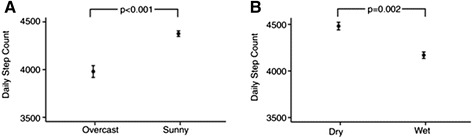
**a** Daily step count on Overcast versus Sunny days. **b** Daily step count on Dry versus Wet days. Data are means ± standard errors of the average for each patient; p-values by paired *t*-test

Physical activity was higher on days with sunshine or without rain (Fig. 3). The mean of patient’s average step count on sunny days was 3938 per day (SD 2447) compared to 3596 per day (SD 2260) on overcast days (paired *t*-test; *p* < 0.0010). Similarly, on dry days the mean of each patient’s average step count was 3999 per day (SD 2507) compared to 3771 per day (SD 2349) on days with rain (*p* < 0.0001).

The day of week effected both daily step count and hours outside. A post-hoc analysis of variance showed that daily step count was 434 steps per day lower on Sunday than Saturday (*p* < 0.001) and 353 steps per day lower on Saturday than Friday (*p* < 0.001). Similarly, time outdoors was 0.55 h lower on a Sunday compared to Saturday (*p* < 0.001) and by 0.09 h lower on Saturday compared to Friday (*p* < 0.001) (see Additional file 1: Figure S1).

### Adjusted analysis

Table 2 shows results from the GEE models with daily step count data recorded during either (a) the whole week and (b) over Monday to Friday (weekdays). Daily step count increased significantly with warmer, sunny weather and fell with wet weather. Over the whole week, higher O_3_ levels were associated with decreased activity (*p* = 0.005) but not with PM_10_ (*p* = 0.112). Conversely, over just weekdays, PM_10_ was associated with reduced activity (*p* = 0.018) but not O_3_ (*p* = 0.239). There were no significant seasonal effects (sine and cosine terms) with temperature included in the model. With inclusion of an autoregressive term, over the whole week, rise in O_3_ was still associated with reduced daily step count (*p* = 0.008) and rise in PM_10_ also significantly and independently associated with reduced daily step count (*p* = 0.047). Inclusion of time outdoors as an independent variable in the regression model, eliminated the effect of O_3_ on daily step count over the whole week (regression coefficient = -3.9; 95 % CI -8.8 to 0.9; *p* = 0.113) and similarly between step count and PM_10_ over weekdays only (regression coefficient = -4.4; 95 % CI -10.4 to 1.5; *p* = 0.147).

**Table 2:** Relationship between daily step count and environmental factors (climate, pollutants and weeksday) over the full week, and during weekdays only; allowance was made for season, linear trend and day-length (data for these variables not shown)

	Over full week			Weekdays only		
	Regression coefficient	95 % CI	p-value	Regression coefficient	95 % CI	*p*-value
Temperature (°C)	37.7	14.1 to 61.4	0.002	36.5	9.6 to 63.4	0.008
Sunshine (% day)	4.7	2.8 to 6.7	<0.001	4.0	1.8 to 6.2	<0.001
Rainfall (mm)	-16.8	-27.1 to -6.6	0.001	-15.3	-26.4 to -4.2	0.007
Wind speed (m/s)	-17.2	-37.6 to 3.2	0.099	-43.8	-66.2 to -21.5	<0.001
PM_10_ (μg/m^3^)	-5.4	-12.2 to 1.3	0.112	-7.8	-14.2 to -1.3	0.018
O_3_ (μg/m^3^)	-8.0	-13.5 to -2.4	0.005	-3.5	-9.4 to 2.4	0.239

Table 3 shows only the effects of the two pollutants (PM_10_ and O_3_) on the various outcome measures over the whole week (Table 3); over week-days (see Additional file 1: Table S1) and over the week-end (see Additional file 1: Table S2).

**Table 3:** Relationship between pollutants (PM_10_ and O_3_) and Daily steps count, hours spent outdoors, health status (CAT score), PEFR, dyspnoea, over the full week including Saturday and Sunday; with allowance for season, linear trend, day-length, temperature, sunshine, rain and wind

	Effect of 1 μg/m^3^ PM_10_			Effect of 1 μg/m^3^ O_3_		
	Regression coefficient	95 % CI	*p*-value	Regression coefficient	95 % CI	*p*-value
Step count	-5.4	-12.2 to 1.3	0.112	-8.0	-13.5 to -2.4	0.005
Hours outdoors	2.3 × 10^-3^	-6.5 × 10^-3^ to 1.8 × 10^-3^	0.275	-9.9 × 10^-3^	-14.2 × 10^-3^ to -5.6 × 10^-3^	<0.001
CAT score	9.8 × 10^-3^	-19.6 × 10^-3^ to 39.1 × 10^-3^	0.515	16.0 × 10^-3^	-14.0 × 10^-3^ to 45.9 × 10^-3^	0.296
PEFR	-0.077	-0.17 to 0.018	0.110	-0.089	-0.18 to 0.001	0.054
Dyspnoea	-0.41 × 10^-3^	-8.0 × 10^-3^ to 7.1 × 10^-3^	0.915	6.6 × 10^-3^	1.3 × 10^-3^ to 12.0 × 10^-3^	0.015

Time spent outdoors fell with higher O_3_ levels (*p* < 0.001) for data collected over the whole week, just weekdays (*p* = 0.001) and at weekends (*p* < 0.001). PM_10_ show no effects on time spent outdoors on either whole week (*p* = 0.275) or weekdays (*p* = 0.217) or weekends (*p* = 0.502). Dyspnoea increased and PEF fell with higher levels of O_3_ over the whole week (*p* = 0.015 and *p* = 0.054 respectively) and for weekdays only (*p*= 0.017 and *p* = 0.040 respectively) but not at weekends. No effects of PM_10_ were observed on daily dyspnoea or PEF. No effects of either pollution were seen on daily CAT score.

**Fig. 4:**
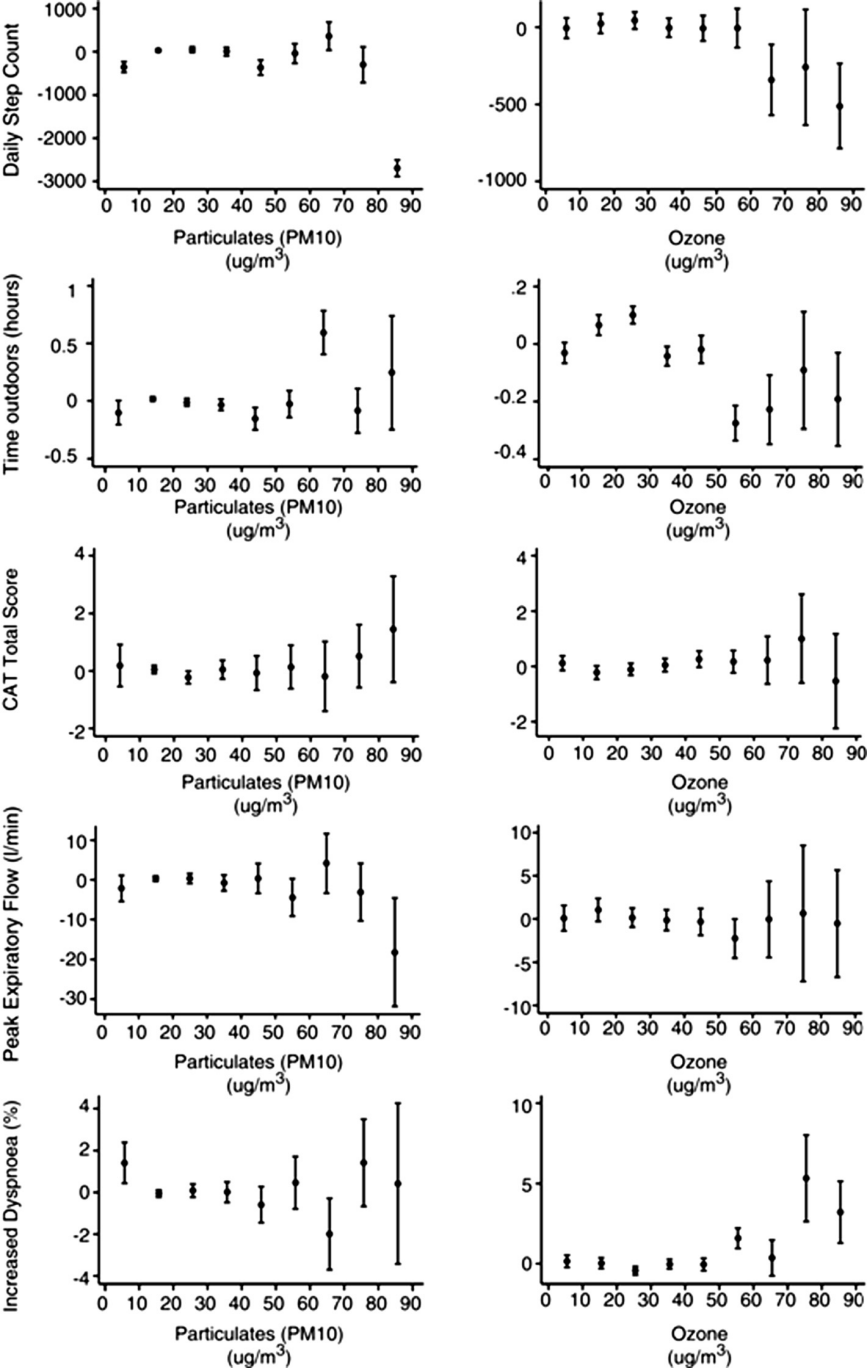
Residuals from a GEE model that included temperature, wind speed, rainfall, hours of sunshine, day length, season and linear trend, plotted against daily PM_10_ and Ozone (O_3_) levels; data are averaged over 10 μg/m^3^ intervals; bars as ± standard error

Figure 4 shows the residuals after fitting the climatic and other variables plotted against PM_10_ and O_3_. The plots show little effect of the pollutants on daily step count, time outdoors, PEFR and dyspnoea until they exceed around 60-70 μg/m^3^. CAT score appears unrelated throughout the range of pollutants.

**Fig. 5:**
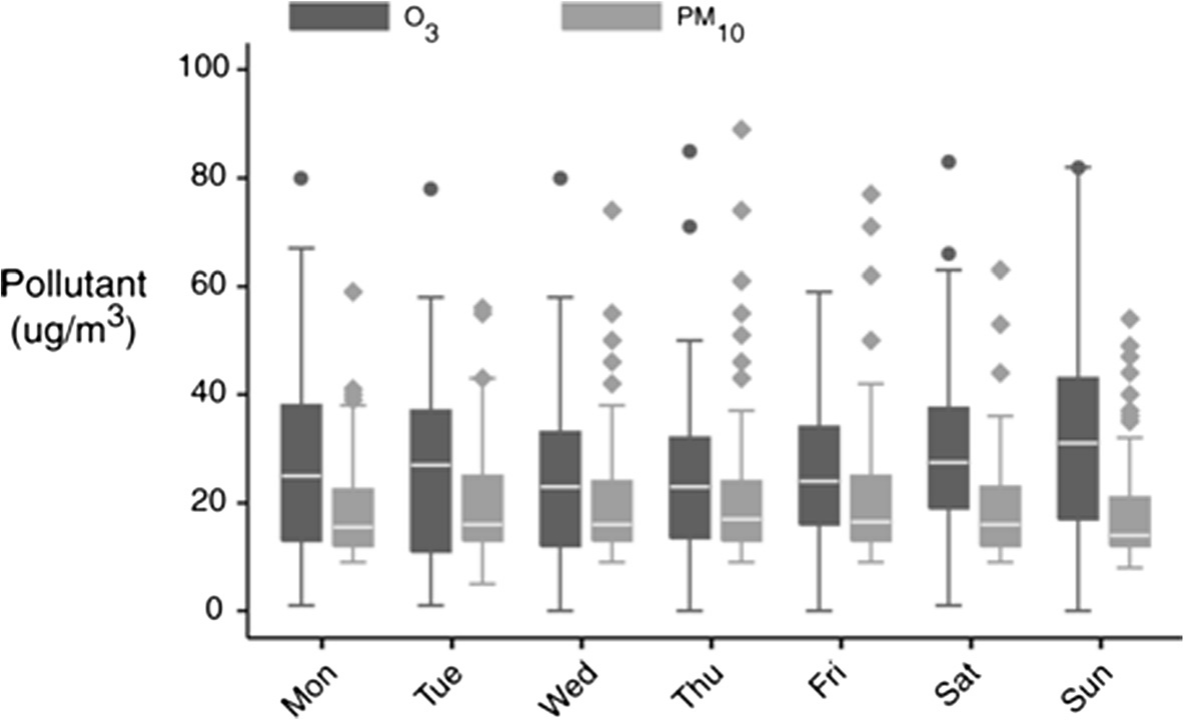
PM_10_ and O_3_ concentrations during the week between 7^th^ April 2011 and 31 March 2013

Figure 5 shows that O_3_ concentration was significantly higher by 4.6 μg/m^3^ and PM_10_ levels 1.73 μg/m^3^ lower during the weekend (*p* = <0.001 and *p* = 0.057 respectively).

## Discussion

This study shows that day-of-the-week, meteorological factors and for the first time that high levels of atmospheric pollutants affect physical activity in COPD patients. The reduction in activity at weekends was not unexpected as this is typically a period of rest. Days that were warm, dry and sunny appeared to encouraged patients to go outside and walk more, whereas cold, rainy and overcast days reduced activity. A number of studies have observed that physical activity decreases in healthy adults during the colder, shorter winter months or increases on longer, sunny days [25]. Indeed, some studies have shown seasonal variation in activity in COPD patients [26–28] and is reduced at weekends compared to weekdays [29, 30] but we extend these findings by showing that activity is primarily related to meteorological conditions irrespective of the season.

Our findings are important because COPD patients already have a reduced exercise capacity due to their airflow limitation. Any further reductions of activity due to the weather or day-of-the-week may worsen muscular de-conditioning which is common in inactive COPD patients. Muscle weakness and feelings of fraility may make the patients feel unable to leave their homes and once this behaviour is established may prove difficult to reverse. It might in part explain why health related quality of life is poorer in winter than spring or summer [31] and measures of anxiety and depression higher in winter [32]. The findings are also important because for the first time we show an effect of atmospheric pollution on physical activity which was only possible because we studied a group whose air-flow limitation is sufficient to make such effects apparent.

There are mechanisms by which outdoor atmospheric pollution might cause patients to be less active when outdoors. O_3_ above 200 ppb can affect peak expiratory flow in elite cyclists during maximal exercise [33] but may not cause problems at low levels [34]. We found that PEF was also reduced only at high levels of O_3_ during the weekdays (*p* = 0.040) though it just failed to reach significance over the whole week (*p* = 0.054). Atmospheric pollutants can also produce harmful effects on the airways, such as pulmonary and systemic inflammation [35, 36], reduction in airway ciliary activity [37], increases in bronchial reactivity [38] and airway oxidative stress [39]. Exposure to O_3_ can also significantly increases heart rate and blood pressure, as well as causing mitochondrial damage [40]. However, whether patients are aware of these systemic and anatomic effects is not clear. We found that dyspnoea increased with higher O_3_ levels but did not find any effect on the CAT quality of life score. Some patients may not have gone outdoors when the pollution levels were high but it is not obvious how the patients knew not to go out. There is little evidence that people alter their behaviour in response to pollutant alerts in the news or from other advisory systems [41]. O_3_ is a colourless, odourless, gas which cannot be seen or smelt but its precursors are mainly motor vehicle exhaust fumes might be detected [41]. In London, a pollution haze can be seen on some days [42] but the patients might not live on hills or in high-rise buildings where these observations can easily be made. High levels of O_3_ are known to be associated with hot weather which might discourage patients from taking exercise. However, we excluded from the analysis the hottest days with mean temperatures over night and day exceeding 22.5 °C. Further studies are needed to determine if and how COPD patients can detect increased atmospheric pollution.

The limitations of this study should be discussed. We were not able to assess the intensity of the physical activity. This can be measured with accelerometers but would require weekly or fortnightly clinic visits by patients to download data which was not practical in this long term study. Pedometers can be inaccurate in slow walking individuals but this would be a consistent bias in a given patient and thus unlikely to alter how they respond to changes in pollution or the weather. Another limitation was that we did not collect pedometry data as fully as the PEF or dyspnoea data and we have no control group. Some patients did not wear their pedometer every day, some were lost and/or broken when inadvertently washed and a replacement only possible at their 3 monthly clinic visit. Some patients were excluded because too little data remained after excluding periods of exacerbation. These excluded patients may well have been frequent exacerbators, and thus our findings might not necessarily apply to this group though the exacerbation frequency in the studied group was similar to the 126 patients not included. We have also not examined other weather conditions such as snow or ice, when the risk of slipping might discourage excursions outdoors. It was not practical to monitor the pollution and climate exposure of each individual and thus we assumed that the pollution levels at the monitoring site in Bloomsbury and weather measured at Heathrow were indicative of that experienced by the patient. Previous studies have shown the data at Bloomsbury is correlated with outer suburban sites [43] and similar in temporal evolution to other sites in London [44]. Although, we did not use personal pollution monitors we did collect individual outcome data – and this semi-individual design is considered valid for air pollution studies [45]. In our analysis, we felt it necessary to analyse separately weekdays and weekends as well as the whole week because the “day-of-the-week” effect was very large and may have confounded the results. In many countries, O_3_ is significantly higher at weekends compared to weekdays [46–48] whereas PM_10_ is higher at weekdays [46]. We found similar effects in London. By analysing the data in this way, we reduced the statistical power and this could explain the absence of consistent effects during both weekdays and weekends.

## Conclusions

There are a number of important implications to this work. Patients with COPD should be encouraged to increase physical activity as pulmonary rehabilitation reduces breathlessness, improves quality of life and exercise tolerance. Inactivity is greatest during cold weather and perhaps pulmonary rehabilitation programmes should be targeted in the winter to limit this inactivity. Activity is also reduced at weekends and patient education should encourage patients to maintain activity on these days. This study provides evidence of an effect on the daily activity of COPD patients of atmospheric pollution at higher levels and public health schemes to reduce levels of atmospheric pollution should be further encouraged.

## Additional file

Additional file 1:
**Influence of weather and atmospheric pollution on physical activity in patients with COPD.**

## Abbreviations

COPD: Chronic obstructive pulmonary disease
PEF: Peak expiratory flow
PR: Pulmonary rehabilitation
FEV1: Forced expiratory volume in one second
FVC: Forced vital capacity
PM10: Particulate matter <10 μm in diameter
BMI: Body mass index
CAT: COPD assessment test
O_3_: Ozone
GEE: Generalised estimating equations
ANOVA: Analysis of variance
GOLD: Global initiative for chronic obstructive lung disease
SEM: Standard error of the mean
IQR: Inter quartile range
SD: standard deviation
