# Supplementary material for: Influence of weather and atmospheric pollution on physical activity in patients with COPD
Source: Respir Res. 2015 Jun 13;16(1):71. doi: 10.1186/s12931-015-0229-z (PMC4470337; doi:10.1186/s12931-015-0229-z)

Influence of weather and atmospheric pollution on physical activity in patients with COPD.

Ayedh D Alahmari[1], Alex J Mackay[1], Anant RC Patel[1], Beverly S Kowlessar[2], Richa Singh[2], Simon E Brill[2], James P Allinson[2], Jadwiga A Wedzicha[2], Gavin C Donaldson[2]

Online Data Supplement

Table S1. Relationship between pollutants (PM_{10} and O_3) and Daily steps count, hours spent outdoors, health status (CAT score), PEFR, dyspnoea, ***over weekdays (Monday-Friday);*** *with allowance for season, linear trend, day-length, temperature, sunshine, rain and wind.*

	**Effect of 1 μg/m^3 PM_{10} (Weekdays)**				**Effect of 1 μg/m^3 O_3 (weekdays)**		
	**Regression Coefficient**	**95% CI**	**p-value**		**Regression Coefficient**	**95% CI**	**p-value**
**Step count**	-7.8	-14.2 to -1.3	0.018		-3.5	-9.44 to 2.35	0.239
**Hours outdoors**	$-2.5x10^{-3}$	$-6.5\ x10^{-3}$ to $1.4x10^{-3}$	0.217		$-7.2x10^{-3}$	$-11.6\ x10^{-3}$ to $-2.9\ x10^{-3}$	0.001
**CAT score**	0.005	-0.02 to 0.03	0.749		0.02	-0.015 to 0.053	0.267
**PEFR**	-0.09	-0.18 to 0.002	0.056		-0.11	-0.23 to -0.005	0.040
**Dyspnoea**	$0.3\ x10^{-3}$	$-8.0\ x10^{-3}$ to $8.7\ x10^{-3}$	0.941		$7.1\ x10^{-3}$	$-1.3x10^{-3}$ to $13.0x10^{-3}$	0.017

Table S2. Relationship between pollutants (PM_{10} and O_3) and Daily steps count, hours spent outdoors, health status (CAT score), PEFR, dyspnoea, ***over the weekend only (Saturday and Sunday)****; with allowance for season, linear trend, day-length, temperature, sunshine, rain and wind.*

	**Effect of 1 μg/m^3 PM$_{10}$ (Weekend)**				**Effect of 1 μg/m^3 O$_3$ (Weekend)**		
	**Regression Coefficient**	**95% CI**	**p-value**		**Regression Coefficient**	**95% CI**	**p-value**
**Step count**	-2.58	-15.97 to 10.8	0.705		-10.7	-20.4 to -0.89	0.032
**Hours outdoors**	-2.6x10^{-3}	-10.2 x10^{-3} to -5.0 x10^{-3}	0.502		-13.1 x10^{-3}	-19.7 x10^{-3} to -6.5 x10^{-3}	<0.001
**CAT score**	0.027	.011 to 0.065	0.157		-0.006	-0.030 to 0.043	0.717
**PEF**	-0.047	-0.20 to 0.11	0.554		0.04	-0.13 to 0.056	0.441
**Dyspnoea**	3.81 x10^{-3}	-16.1 x10^{-3} to 8.45 x10^{-3}	0.542		-4.27 x10^{-3}	5.32x10^{-3} to 1.38 x10^{-3}	0.383

Figure S1. Daily Step count and Time outside during the week in COPD patients; data are means + standard errors for daily step count; median + inter-quartile range for hours outside; p-values from a post-hoc analysis of variance and wilcoxon rank-sum test respectively.

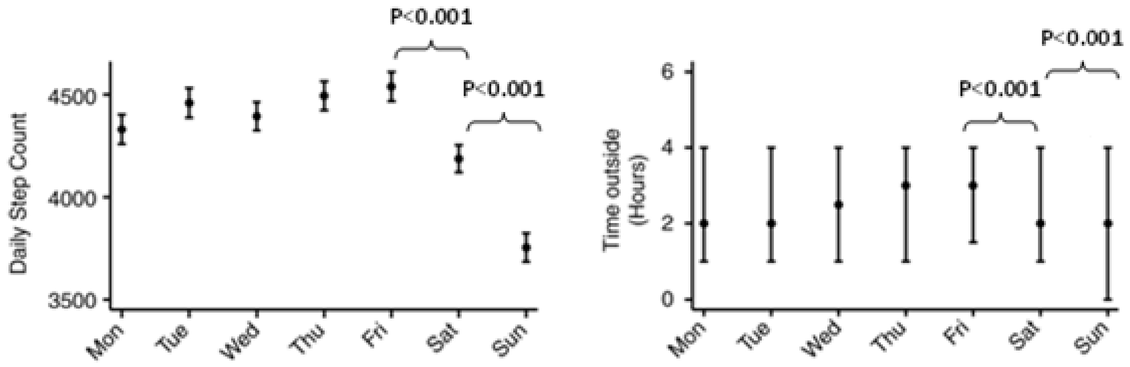

Supplement: Additional file 1: — Influence of weather and atmospheric pollution on physical activity in patients with COPD. [file 12931_2015_229_MOESM1_ESM.pdf]
